# Tumefactive Demyelination: A Common Radiological Masquerader of Malignancy and the Expeditious Diagnostic Applicability of Crush Smear Cytology

**DOI:** 10.7759/cureus.29751

**Published:** 2022-09-29

**Authors:** Rajaguru Paramaguru, Subramaniam Ramkumar

**Affiliations:** 1 Pathology, Al Salam International Hospital, Kuwait City, KWT; 2 Pathology, PVS (PV Swami) Memorial Hospital, Ernakulam, IND; 3 Pathology, Woodland Hospital, Shillong, IND

**Keywords:** brain malignancy, gliomas, crush cytology., brain biopsy, tumefactive demyelination

## Abstract

Tumefactive demyelinations (TDs) are demyelinating central nervous system lesions that masquerade as neoplastic lesions on radiological images. Brain biopsy is often required for confirmatory diagnosis. Since crush cytology has become a routine practice, a thorough knowledge of the cytomorphologic features of TD is required to prevent misdiagnosis. In this report, we describe the cytomorphological and histomorphological features of a case of TD.

## Introduction

Tumefactive demyelinations (TDs) are well-demarcated lesions mimicking neoplasm on radiological images. On magnetic resonance imaging (MRI), these lesions present as space-occupying lesions with irregular infiltrative borders showing peri-lesional edema, and necrosis with cystic degeneration resembling an axial malignancy. Hence, a brain biopsy and histopathological examination are mandatory for diagnosing such lesions [1]. Histomorphologic or cytomorphologic examinations of these lesions should be interpreted with a high index of suspicion of TD because the presence of highly cellular sections, reactive nuclear atypia in astrocytes, and increased mitotic activity can lead to an erroneous diagnosis of a glial neoplasm. An early intraoperative diagnosis of TD can be made on crush smears [2] but the cytological features of crush smears have not received importance. Also, good knowledge about the cytological features of crush smears of TD is mandatory to exclude various neoplastic and non-neoplastic conditions to avoid misdiagnosis. In this report, we describe the distinctive crush smear cytomorphological features and histological features of a case of TD.

## Case presentation

A 52-year-old female presented with a history of headache and short-term memory disturbance associated with disorientation to space and person which was of insidious onset and progression. She also had intermittent nausea and spells of dizziness. Mild impairment of sensory disturbances in the right upper and lower limb was seen. 

MRI revealed left frontoparietal well-defined hyperintense lesion measuring 2.9 × 2.7 × 2.5 cm with mild surrounding edema and incomplete peripheral enhancement. MR spectroscopy revealed increased choline/creatine ratio and increased choline/N-acetyl-aspartate (NAA) ratio with lactate peak. The final impression was low-grade glioma. Frontal craniotomy was performed and two tiny biopsy fragments were given for intraoperative diagnosis. The tissues measured 0.8 × 0.5 × 0.2 cm and 0.5 × 0.4 × 0.2 cm each. Tiny bits from different areas of the tissue were taken and crush smears were made. Slides were fixed in 95% ethanol and stained with hematoxylin and eosin stain.

**Figure 1:**
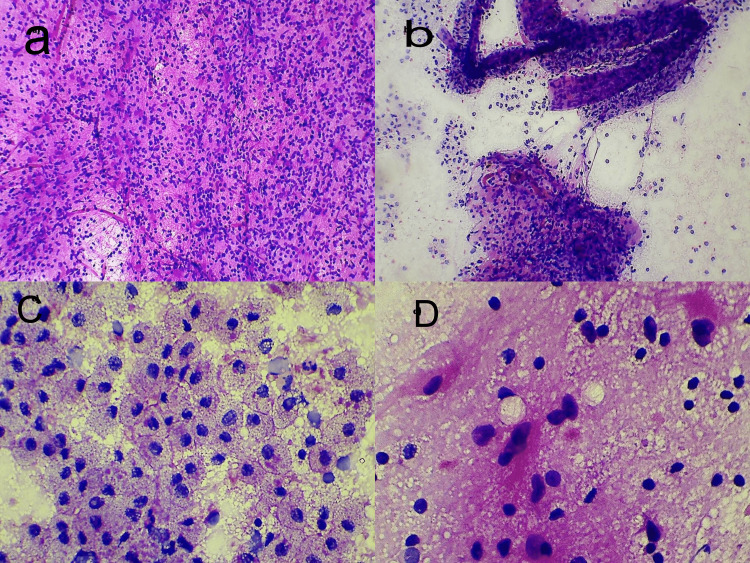
Cytomorphological features of tumefactive demyelination (crush cytology). a) Cellular smear showing numerous scattered mature lymphocytes and reactive astrocytes in fibrillary background. Fine vascular fragments are also noted (crush cytology, H&E 100×). b) Vascular fragments showing dense perivascular lymphohistiocytic cuffing (crush cytology, H&E 100×). c) Sheets of foamy histiocytes scattered throughout the smear (crush cytology, H&E 400×). d) Closer view of the reactive astrocytes with nuclear atypia and a fibrillary cytoplasm (crush cytology smear, H&E 400×). H&E, hematoxylin and eosin.

Microscopic examination revealed varied cytomorphology on the slides. Few slides studied from the lesion showed scattered neurons embedded in fibrillary matrix, representing the normal gray matter. Slide from the lesional area showed numerous scattered foamy histiocytes and many vascular fragments with perivascular aggregates of mature lymphocytes and histiocytes. Numerous scattered reactive astrocytic cells with moderate nuclear atypia, prominent nucleoli, and moderate eosinophilic cytoplasm with processes were noted. Mitosis and necrosis were absent (Figure 1).

**Figure 2:**
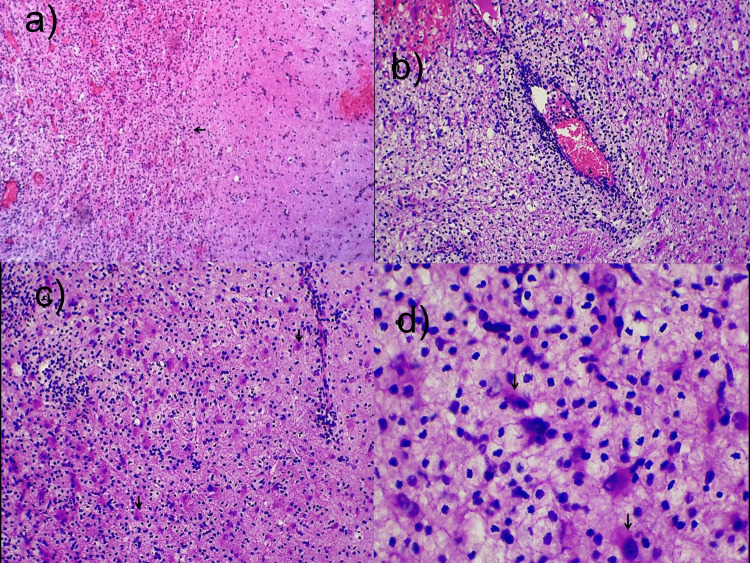
Histomorphological features of tumefactive demyelination a) Tissue section showing well-demarcated lesion with a cellular lesion involving the white matter (tissue section, H&E 40×). The gray matter showing the peripheral rim of lymphohistiocytic cells (black arrow). b) Low-power image shows the perivascular lymphohistiocytic collection (tissue section, H&E 100×). c) Low-power image shows the reactive astrocytes (black arrows) and histiocytes (tissue section, H&E 100×). d) High-power image shows the reactive astrocytes (black arrows) and histiocytes (tissue section, H&E 400×). H&E, hematoxylin and eosin.

The histological sections showed a well-demarcated lesion predominantly involving the white matter. The lesion consisted of sheets of histiocytes, mature lymphocytes, and reactive astrocytes similar to features on crush smears. Perivascular lymphohistiocytic cells were seen (Figure 2).

## Discussion

Crush smear cytology is routinely performed for rapid intraoperative diagnosis of central nervous system tumors and the cytomorphological features of these lesions are well known [3]. The procedure is simple, cost-effective, and user-friendly. The results can be produced in a timely manner with minimal use of resources. The diagnostic utility and efficacy in guiding neurological resection have been previously proved by various studies. However, cytological features of pseudoneoplasms like TD have been given less importance. Crush smears can be very useful in the rapid diagnosis of TD even with stereotactic biopsies [2]. Neelima R, et al. [4] have also shown similar cytomorphological features in their cases. Of their six cases of TD, four cases were misdiagnosed as gliomas. These cases showed abundant foamy macrophages in sheets without necrosis, perivascular lymphocytic infiltrates, and aggregates of abundant foamy macrophages. Astrocytes with reactive nuclear atypical changes were seen and were misinterpreted as tumor cells. Two cases were offered a diagnosis of TD due to the absence of nuclear pleomorphism. 

Hence, the knowledge of the cytological features of TD will also help to rule out other malignant and infective conditions which are the causes of misdiagnosis on crush cytology. The differential diagnosis for TD on crush smears includes low-grade and high-grade gliomas, lymphoma, infarction, and infections. Low-grade gliomas show cellular smears composed of oval- to spindle-shaped astrocytes with fibrillary cytoplasm and there is the absence of histiocytes and mature lymphocytes [2,5]. High-grade gliomas show nuclear atypia and mitosis which may be encountered in TD; in addition, they show necrosis and endovascular proliferation [2,5]. Lymphomas show numerous atypical lymphoid cells with or without necrosis [5]. Infective lesions such as progressive multifocal leukoencephalopathy show large basophilic intranuclear inclusions in oligodendrocytes in addition to collections of foamy histiocytes [5]. Infarction shows necrotic tissue and the absence of perivascular lymphohistiocytic collections [5]. In the preparation of crush smears, it is ideal to take multiple tiny bits from the biopsy specimen so that the lesional area is adequately sampled as the reactive astrocytes in the periphery of the lesion may mimic a glioma. It is also mandatory to exclude the presence of necrosis, viral inclusions, granulomas, or organisms before arriving at a conclusive opinion. Necrosis and mitosis are uncommon in TD (since they are reactive lesions) and may pose problems in diagnosis on crush cytology. In such cases, it is better to wait for the histopathological sections.

## Conclusions

A correct diagnosis of TD is a challenge and crush cytology has to be interpreted with a high index of suspicion. Early and prompt treatment of these patients would ensure a remarkable and complete clinical and symptomatic recovery. Awareness about the lesion and a combined diagnostic modality approach between the radiologist, pathologist, and neurologist will ensure prompt and early diagnosis of this lesion. One should consider including this entity in the differential diagnosis of all solitary space-occupying lesions. The presence of numerous histiocytes, reactive lymphocytes, and reactive astrocytes on crush smears should raise a suspicion of TD. Though challenging, crush smear cytology is a useful tool for rapid and cost-effective diagnosis of TD like other CNS neoplasms. Since crush smear cytology has become a routine practice for neuropathologists, a thorough knowledge of the cytological features is mandatory to avoid misdiagnosis.
